# Effect of Dairy Protein Intake on Muscle Mass among Korean Adults: A Prospective Cohort Study

**DOI:** 10.3390/nu12092537

**Published:** 2020-08-21

**Authors:** Eunjin So, Hyojee Joung

**Affiliations:** 1 Department of Clinical Nutrition, The Catholic University of Korea Seoul St, Mary’s Hospital, Seoul 06591, Korea; ajuilmn@naver.com; 2 Department of Public Health, Graduate School of Public Health & Institute of Health and Environment, Seoul National University, Gwanak-gu, Seoul 08826, Korea

**Keywords:** muscle mass, dairy products, milk, middle-aged, Korean Genome and Epidemiology Study (KoGES), cohort study

## Abstract

This cohort study aimed to identify the associations of dairy protein intake with the risk of developing a low muscle mass during a 12-year follow-up period, using data from 4412 middle-aged Korean Genome and Epidemiology Study participants with a normal baseline muscle mass. Dairy protein intake at baseline was assessed using a semi-quantitative Food Frequency Questionnaire. Skeletal muscle mass index (SMI), defined as the weight-adjusted skeletal muscle mass, was measured biennially using multi-frequency bioelectrical impedance analyses. Cox proportional hazards regression analysis was used to calculate multivariable-adjusted hazard ratios (HRs) and 95% confidence intervals (CIs). Overall, 395 subjects developed a low SMI (%) during an average follow-up of 141 (19–152) months. The average consumption of milk and other dairy products was 73.6 and 104.1 g/day, respectively. In men, a higher dairy protein intake was associated with a decreased risk of developing a low SMI (tertile 3 [T3] vs. T1, HR: 0.63; 95% CI: 0.42, 0.94; *p* for trend = 0.029). In a stratified analysis according to a total protein intake, this association was stronger in the lower-protein intake group (HR: 0.59; 95% CI: 0.35, 0.99; *p* for trend = 0.036) but not detected in the higher-protein intake group. Men who consumed milk ≥1 time/day had a significantly lower risk of developing a low SMI (HR: 0.62; 95% CI: 0.39, 0.98; *p* for trend = 0.023). No significant associations were observed in women. In summary, dairy consumption appears to be beneficial for decreasing the risk of developing a low muscle mass in middle-aged Korean men.

## 1. Introduction

Sarcopenia, a syndrome characterized by low levels of muscle mass and strength, is common among older adults but can also occur in younger adults [1]. Sarcopenia is known to be associated with increased risks of falls and fractures, reduced cardiopulmonary function, metabolic syndrome, insulin resistance, and eventually, disability, hospitalization, and death [2]. The current global project estimates that the number of humans aged ≥60 years will increase from 900 million in 2015 to 2 billion in 2050 [3]. In other words, the population of elderly individuals who will be exposed to the risks of deteriorating muscle strength and mobility associated with muscle loss is expected to increase enormously during the next few decades [4]. 

Although sarcopenia is largely attributed to aging, the condition can be accelerated by modifiable lifestyle factors such as physical inactivity, alcohol consumption, smoking, and malnutrition [5]. Among dietary factors, it is generally accepted that adequate dietary protein is required for the maintenance of muscle mass in older adults [6]. Especially, increasing attention has focused on dairy foods, with evidence that essential amino acids play the predominant role in promoting positive muscle protein balance [7]. Although previous studies have suggested a beneficial role of dairy consumption with respect to some types of cancers [8,9], cardiovascular disease [10], metabolic syndrome [11,12], cognitive ability [13,14], bone density [15,16,17,18,19], disability [20], and fracture [21], the role of dairy consumption in preventing muscle mass loss has not been fully investigated. Further, most previous studies were randomized controlled trials focused on milk protein supplementation in combination with resistance training [22]; community-based observational studies, in particular, are lacking and those that have been published reported inconsistent results. For example, one cross-sectional study of Australian women aged 70–80 years found positive associations of dairy intake with lean body mass and physical performance, suggesting that dairy consumption may be a factor in preventing age-related loss of muscle mass [23]. However, a cross-sectional study conducted in the Netherlands reported that the intake of fish/meat/eggs, but not that of plant and dairy protein was positively associated with creatinine excretion, which is a reliable measure of muscle mass [24]. Some clinical intervention studies on dairy proteins, such as ricotta cheese and milk, failed to demonstrate an effect of these products on muscle mass and strength [25,26]. To date, only one prospective study reported an inverse association between dairy consumption frequency and the risk of developing low muscle mass in Korean adults [27].

Milk and other dairy products are nutrient-dense and supply high concentrations of many nutrients, including calcium, phosphorus, vitamin A, vitamin D, riboflavin, vitamin B12, and protein, relative to their overall energy values [28]. Specifically, milk proteins (whey and casein) are nutritionally complete and highly digestible, with high amino acid scores, and contain all amino acids in amounts sufficient to support the maintenance of all body proteins [29,30]. Due to its nutrient profile, the dietary guidelines of most Western countries recommend at least 2–3 daily servings of milk or milk products [15,31,32,33]. The Korean Nutrition Society recommends that Korean adults consume at least one serving of milk per day [34]. However, dairy products are generally not included in the traditional Korean diet that is mainly composed of rice and vegetables [35,36]. As a result, the average individual milk consumption in Korea, 60 g/day [36], is lower than those reported in Western countries such as Sweden (240 g/day) [37] and Spain (206 g/day) [21]. In addition, a recent study in an elderly Korean population revealed that the main sources of dietary protein in the group with the lowest tertile of protein intake were rice and other grains (41.8% in men and 44.1% in women) [38]. As dairy products are sources of animal protein that do not require cooking and can be consumed simply in a liquid form, their consumption may be a practical option for preventing sarcopenia in Korean adults obtaining proteins mainly from plant sources.

Therefore, in this study, we investigated the association of dairy protein intake with the risk of developing a low muscle mass in a population of middle-aged Korean adults using data from the Korean Genome and Epidemiology Study (KoGES).

## 2. Materials and Methods 

### 2.1. Study Population

This study used data from the KoGES, a community-based prospective cohort study of 10,030 adults aged 40–69 years who had lived in urban (Ansan) and rural (Ansung) areas of Korea for at least 6 months before enrollment. Participants were enrolled during 2001–2002 and followed biennially through 2013–2014. In this study, we excluded participants who did not complete a baseline Food Frequency Questionnaire (FFQ) or had incomplete anthropometric data (*n* = 2417), reported implausible energy intake (<500 or >5000 kcal/day; *n* = 56), did not participate in follow-up examinations (*n* = 3042), or had a low skeletal muscle mass index (SMI) at baseline (*n* = 103). The final analysis included 4412 participants. All study procedures were approved by the Institutional Review Board of the Catholic Medical Center (No. KC17ZESI0645). Written informed consent was obtained from all participants.

### 2.2. Assessment of Dairy Consumption

Dietary intake was assessed at baseline using a 103-item, semi-quantitative FFQ. The validity and reproducibility of the FFQ were evaluated elsewhere in detail [39]. Nine response options were provided to identify the frequency of consumption of each food (never or almost never, 1 time/month, 2–3 times/month, 1–2 times/week, 2–3 times/week, 3–4 times/week, 5–6 times/week, 1 time/day, 2 times/day, or 3 times/day), and three response options were provided for portion size (1/2 serving, 1 serving, and ≥2 servings). Dairy products included milk, yogurt, and cheese. We transformed the quantity of consumed dairy products (g/day) into servings/day by dividing the total amount of each consumed food by the standard serving size (200 mL of milk, 130 mL of yogurt, and 20 g of cheese). Daily nutrient intake was calculated based on the seventh edition of the Food Composition Table in Korea [40]. Participants were divided into the lower- and higher-protein intake groups according to a total protein intake of <1.0 and ≥1.0 g/kg body weight/day, as per recommendations of the PROT-AGE study group (to review dietary protein needs with aging), respectively [41]. The consumption frequencies of milk and yogurt were categorized into three groups (<1 serving/week, 1–6 servings/week, and 1 serving/day). 

### 2.3. Covariates

The participants’ demographic and general information and medical histories were gathered using a self-administered questionnaire that included items on sex, age, marital status, education level, income, smoking status, alcohol consumption, physical activity, self-perceived dental health status, chronic disease, and residential area. Marital status was categorized into married and unmarried (including divorced, separated, and others). Education level was categorized into high school or lower and college or higher. Smoking status was categorized into smokers (current smokers) and non-smokers (former smokers and non-smokers). Alcohol consumption was categorized into alcohol drinkers (current drinkers) and non-drinkers (former and never drinkers). Regular physical activity was recorded as “yes” if the participant performed ≥2.5 h of exercise per week, according to the World Health Organization’s recommendation [42,43]. The participants also reported the number of hours spent sleeping and the frequency of activities performed at five intensity levels (sedentary, very light, light, moderate, and heavy activities). Metabolic equivalents (METs)-hours per day were then calculated by multiplying the activity hours per day by the MET value of each type of activity [44]. Self-perceived dental health status was categorized into poor and other (good and fair). The presence or absence of chronic diseases, such as myocardial infarction, congestive heart failure, coronary artery disease, peripheral arterial disease, cerebrovascular disease, asthma, chronic obstructive pulmonary disease, cancer, dementia, and arthritis, was recorded. 

### 2.4. Assessment of Body Composition

The fat mass and fat-free mass values were determined using a multi-frequency bioelectrical impedance analysis machine (MF-BIA, Inbody 3.0, Biospace, Seoul, Korea) with eight-point tactile electrodes. Skeletal muscle mass was estimated by dividing the total lean body mass by 0.52 [45]. In our study, low muscle mass was defined using the SMI, which was calculated as the total skeletal muscle mass (kg)/weight (kg) × 100 to adjust for the participant’s stature and non-skeletal muscle tissue (fat, organs, and bone) mass, as described by Janssen et al. [46]. Low muscle mass was defined as an SMI of <2 standard deviations below the sex-specific normal mean for a young reference group, as defined in a previous Korean study that reported cutoff points of 35.71% for men and 30.70% for women [47]. The incidence of low muscle mass was determined based on the SMI data collected at baseline and every 2 years of follow-up. 

### 2.5. Statistical Analyses 

Dairy protein intake at baseline was categorized into tertiles, and the lowest tertile was used as the reference in the analyses. Within each sex, differences in general characteristics according to the tertiles of dairy protein intake at baseline were examined using the chi-square test for categorical variables and a linear regression analysis for continuous variables. For each participant, the person-time was measured from the date of enrollment in the cohort to the date of a low SMI diagnosis or the final follow-up survey. To assess the association between dairy protein intake and the development of a low SMI during follow-up, we calculated hazard ratios (HRs) and 95% confidence intervals (CIs) using Cox proportional regression models adjusted for potential confounders such as age (years), skeletal muscle mass at baseline (kg), energy-adjusted protein intake (g/day), vitamin intake, marital status, education level, income, smoking status, regular physical activity, self-perceived dental health status, chronic diseases, and residential area. The energy-adjusted protein intake was calculated using the nutrient residual model [48]. Participants were divided into the lower- and higher-protein intake groups, as defined in Section 2.2. The dietary intake of each vitamin (vitamins A, C, and E) was categorized into quartiles. The first quartile received a score of 1; the second, third, and fourth quartiles received scores of 0; and the sum of the scores was calculated for each vitamin [49]. A linear trend was estimated using the median value of each tertile as a continuous variable. The *p*-values for interactions were obtained using the likelihood ratio test using models with and without the interaction terms (total protein intake × dairy protein intake). IBM SPSS Statistics for Windows, version 24.0 (IBM Corp., Armonk, NY, USA) was used for all statistical analyses. A two-sided *p*-value of <0.05 was considered statistically significant. 

## 3. Results

A total of 395 subjects (9.0%) developed a low SMI during an average follow-up of 141 months (range: 19–152 months). The general characteristics of the study participants are presented in Table 1. At baseline, men and women with higher dairy protein intakes were more likely to be younger, live in an urban area, have higher education and household income levels, and to be physically inactive (all *p* < 0.001). The proportion of current smokers was lower among men with a higher dairy protein intake than among those with a lower dairy protein intake (*p* < 0.001). Women with a higher dairy protein intake were less likely to be current alcohol drinkers (*p* = 0.002) and to have a chronic disease (*p* < 0.001). Regarding body composition, men with a higher dairy protein intake had a higher lean mass at baseline (*p* = 0.010). Women with a higher dairy protein intake had a lower fat mass (*p* = 0.024) and a higher SMI (%) at baseline (*p* < 0.001). 

The average consumption of milk and other dairy products was 73.6 and 104.1 g/day, respectively. Men and women in the highest tertile of dairy protein intake reported average milk consumption amounts of 161.1 and 194.2 g/day, respectively; they also showed higher intakes of total energy, energy from fat, and energy from protein as well as a lower intake of energy from carbohydrates (all *p* < 0.001; Table 2). Additionally, both men and women with a higher dairy protein intake had higher intakes of vitamin A, C, and E after adjusting for age and total energy intake (all *p* < 0.001).

Table 3 presents the HRs and 95% CIs for the risk of developing a low SMI according to the tertile of dairy protein intake at baseline. After adjusting for covariates, men in the highest tertiles of dairy and milk protein intakes had a significantly lower risks of developing a low SMI, compared to those in the lowest dairy (HR: 0.63; 95% CI: 0.42, 0.94; *p* for trend = 0.029) and milk tertiles (HR: 0.66; 95% CI: 0.45, 0.96; *p* for trend = 0.048). In contrast, these associations were not observed in women. In a further analysis of men according to the total protein intake level, this association was found to be stronger in the lower-protein intake group (HR: 0.59; 95% CI: 0.35, 0.99; *p* for trend = 0.036) but was not detected in the higher-protein intake group (*p* for interaction = 0.004). 

The associations between milk and yogurt consumption frequencies and the incidence of low SMI are presented in Table 4. After adjusting for covariates, men in the highest milk consumption group (≥1 serving/day) exhibited a decreased risk of developing a low SMI, compared to those in the lowest milk consumption group (HR: 0.62; 95% CI: 0.39, 0.98; *p* for trend = 0.023). However, no significant association was observed in women. There was no association between yogurt consumption frequency and the incidence of low SMI in men or women.

## 4. Discussion

In this prospective study of a middle-aged Korean population, a higher dairy protein intake was associated with a decreased risk of developing a low SMI in men but not in women, and this association was stronger among men with a low total protein intake. In addition, the consumption of more than one serving of milk per day was significantly associated with a lower risk of developing a low SMI among men. 

We observed an inverse association between dairy protein intake and the risk of developing a low SMI among Korean men after adjusting for covariates. This finding is consistent with the results a study conducted in Korea [27] as well as of those conducted in other countries [23,50]. The previous Korean study, including three different population-based cohorts, reported that the consumption of ≥1 servings of milk per day was significantly associated with a higher SMI and muscle strength [27]. In a cross-sectional study of elderly Australian women [23] and a randomized clinical trial of elderly Mexicans [50], dairy protein intake had a significant positive effect on muscle mass. In contrast, several other studies failed to observe such associations. In a cross-sectional study of an adult cohort with a wide-age range in the Netherlands, dairy intake was not associated with creatinine excretion, an indicator of muscle mass [24]. The authors of that study explained that the lack of such an observation suggests that even the lowest level of dairy protein intake (13.6 g/day) might have already maximally benefitted the muscle mass in this population. A recent European cohort study of community-dwellers aged ≥60 years also identified a null association between habitual dairy consumption and the risk of falling due to low muscle mass [21]. Another systematic review of 14 randomized controlled trials involving 883 adults aged 18–85 years reported that increased dairy consumption resulted in a greater gain in lean mass only in people administered energy-restricted diets [51]. In addition, a recent meta-analysis of 14 randomized controlled trials involving 1424 participants aged 61–81 years showed that dairy protein intake at a level of 14–40 g/day enhanced gains in muscle mass (0.13 kg; 95% CI: 0.01,0.26; *p* = 0.04), but not in handgrip or leg press strength [22]. 

Among men in our study, the association between dairy consumption and a low SMI was stronger in the lower-protein intake group but not detected in the higher-protein intake group. Evidence that dairy supplementation is more effective in individuals with a low protein intake is limited so far, however, some studies have reported that dairy supplementation did not have additional benefit during resistance training in individuals with adequate protein intake [52,53]. In one of these studies, casein hydrolysate protein supplementation was shown to not further enhance skeletal muscle mass and strength gain after prolonged resistance-type exercise training in healthy elderly men who habitually consume adequate amounts of dietary protein (1.1 g/kg/day) [52]. A previous review similarly concluded that increasing protein intake did not enhance resistance training-induced improvements in muscle mass and strength in older individuals who consumed adequate amounts of dietary protein (≥0.8 g/kg/day) [54]. Thus, the lack of a significant association in the higher-protein intake group in men may be explained by the results that additional protein supplementation was meaningless to those who already consumed adequate protein amounts. Another explanation for this finding might be that dairy products supplied other micronutrients needed for muscle maintenance in the lower-protein intake group. Specifically, dairy products contribute substantial amounts of many essential vitamins and minerals as well as protein which play an important role in supporting muscle mass preservation through several physiological mechanisms [18,55]. Consistent with this hypothesis, a previous analysis of data from the United States National Health and Nutrition Examination Survey 2003–2006 showed that adding 1 serving of dairy products per day to a person’s diet significantly improved the intake of 3 nutrients of concern (calcium, vitamin D, and phosphorus) [56]. The traditional Korean diet is composed of rice and vegetables and very little meat and milk [57]. Recently, as the traditional Korean diet has been changing to a Western-style diet, the consumption of animal products, including meat as well as milk and dairy products, has increased. The actual meat intake among Koreans has gradually increased from 67.8 g/day in 1995 to 102.4 g/day in 2015 [36]. Nevertheless, dairy consumption remains relatively low compared to that in Western populations. A recent study reported that the proportion of Koreans with a milk consumption of <1 serving size daily was 83.5% in men and 76.7% in women [11]. In addition, according to data from the 2013–2014 Korean National Health and Nutritional Examination Survey, approximately 47.9% of men and 60.1% of women older than 60 years consumed less than the recommended nutrient intake of protein (0.91 g/kg/day) [58]. Moreover, among Korean adults with a low protein intake, more than 70% of the protein was derived from plants [38], so eating dairy products may improve the quality and quantity of protein as well as supplement other nutrients, which will help their muscle health. 

Currently, many countries have issued guidelines recommending the intake of at least one serving of dairy products per day [15,59], which is consistent with the findings of our study. In men, the highest milk consumption frequency (≥1 serving/day) was associated with a lower risk of developing a low SMI, compared to the lowest milk consumption frequency. In most previous Korean studies, the consumption of ≥1 serving of milk per day decreased the risks of developing a low skeletal muscle mass [27] and metabolic syndrome [11,13]. In a study of Australian women, those in the highest tertile of dairy consumption (≥2.2 servings/day) had a significantly greater whole lean mass and appendicular skeletal muscle mass than those in the lowest tertile (≤1.5 servings/day) [23]. In a Spanish cohort study of community-dwelling adults aged ≥60 years, participants who consumed 1 serving per day of low-fat milk and yogurt had a lower incidence of frailty than those who consumed <1 serving per week [60]. A randomized controlled trial of younger adults with obesity aged 18–50 years consuming a balanced calorie-deficit diet (− kcal/day) demonstrated better maintenance of lean body mass in participants who consumed a diet supplemented with 3 servings of yogurt than in those in the placebo control group (<1 serving/day) [61]. Additional high-quality, large-scale, randomized controlled trials with longer follow-up periods are needed to establish guidelines for dairy protein intake that reflect the subjects’ characteristics according to age, sex, and obesity status as well as protein intake levels. 

Our results showed that dairy protein intake was associated with a decreased risk of developing a low SMI in men, but not in women. Contrary to our results, most previous studies on the association between dairy consumption and muscle mass did not show differences between men and women [24,27,50,61]. In addition, a Swedish cohort study reported a stronger association between milk intake and fractures in women than in men [37]. Our findings may be partly attributable to confounding factors, particularly alcohol consumption, which has previously shown negative effects on muscle mass. According to a recent cross-sectional study, among elderly women, binge drinkers with a weekly or daily consumption had a 3.9 times higher prevalence of sarcopenia than social drinkers [62]. In our study, women with a higher dairy protein intake were more likely to be current drinkers than those with a lower dairy protein intake; this may have attenuated the association between dairy protein intake and the risk of developing a low SMI. Differences in age-related physiological changes in muscle mass between men and women might also explain our results. According to a study of Korean individuals aged ≥10 years who participated in the 2008–2012 Korean National Health and Nutritional Examination Survey, men experience a linear decrease in muscle mass that begins at the age of 30 years, while women show a slow increase in muscle mass until their 40s, a plateau state until the age of 50–60 years, and a decrease thereafter [63]. Since the pattern of muscle mass reduction is relatively flat in middle-aged women, the effect of dairy product intake on muscle mass may not be apparent. 

We used antioxidants intake (vitamin A, C, and E) as a covariate when calculating the multivariable-adjusted HR. Oxidative stress and the accumulation of reactive oxygen species potentially contribute to age-related muscle loss [64]. Although few interventional and observational studies have investigated the association between antioxidants intake and sarcopenia [49,65,66], antioxidant nutrients have been suggested as a nutritional risk factor of sarcopenia. 

The present study had several limitations. First, BIA, a non-invasive method for skeletal muscle mass assessment, is useful in large population-based studies. However, the results may be affected by several factors, including age, hydration status, food or beverage consumption, and exercise intensity. To reduce the possibility of measurement errors, the participants were requested to fast before the BIA, and their hydration status was monitored carefully. Second, we assessed dietary intake only at baseline and did not determine whether these variables had changed over time. Third, we used the FFQ, which cannot measure absolute intake, to estimate the participants’ usual dietary intakes. However, this tool is useful in categorizing individuals on the basis of relative intakes and is the most commonly used for assessing nutrient intakes in epidemiological studies. Fourth, we could not examine the effect of cheese consumption on the development of a low SMI because 85% of the participants never or rarely consumed cheese. Finally, this study categorized total protein intake into two groups owing to the small sample size. Further large-scale prospective cohort studies to elucidate these issues are necessary.

## 5. Conclusions

In conclusion, our findings indicate that higher dairy protein intake decreased the risk of developing a low SMI among Korean men, but not women. This association was stronger among men with a low total protein intake. In addition, consuming more than one serving of milk per day significantly decreased the risk of developing a low SMI among Korean men. These results suggest that dairy consumption may be a modifiable lifestyle factor that can help prevent muscle mass loss, especially in adults with an insufficient total protein intake. 

## Figures and Tables

**Table 1:** General characteristics of study participants according to tertiles of dairy protein intake ^a^ at baseline.

	Men (*n* = 2096)							Women (*n* = 2316)						
Variables	T1		T2		T3		*p* for Trend ^b^	T1		T2		T3		*p* for Trend
	Mean	SD	Mean	SD	Mean	SD		Mean	SD	Mean	SD	Mean	SD	
Age (years)	50.9	8.2	49.1	7.5	50.4	7.9	<0.001	52.8	9.0	49.4	7.9	50.6	8.1	<0.001
Residence area (% urban)	58.9		70.8		74.9		<0.001	42.8		68.8		69.4		<0.001
Education (% ≥College)	18.8		27.8		29.8		<0.001	3.7		10.0		10.1		<0.001
Household income (% ≥3,000,000 KRW)	22.0		29.9		30.6		<0.001	10.7		20.6		19.5		<0.001
Marital status (% married)	96.9		96.8		97.3		0.640	86.1		90.1		87.6		0.380
Dental health status (% poor)	39.9		37.2		38.8		0.677	45.8		39.0		40.9		0.051
Chronic disease (% yes)	1.8		1.0		1.4		0.499	6.8		4.0		3.0		<0.001
Physical activity (METs-hours/day)	23.9	15.5	21.8	13.4	22.5	13.4	0.020	24.4	15.1	20.9	12.9	21.7	13.1	0.021
Alcohol consumption (% yes)	72.3		76.4		72.8		0.819	22.7		26.8		29.5		0.002
Smoking (% yes)	50.0		43.8		40.1		<0.001	2.9		1.6		2.5		0.633
BMI (kg/m^2^)	24.4	2.8	24.5	2.8	24.3	2.6	0.321	24.8	3.0	24.7	2.9	24.4	2.9	0.356
Fat mass (kg)	15.1	4.7	15.2	4.5	14.7	4.4	0.069	18.7	4.9	18.6	4.5	18.4	4.6	0.024
Lean mass (kg)	52.9	6.4	53.9	6.1	53.2	5.9	0.010	39.9	4.6	40.5	4.2	40.1	4.1	0.218
SMI (%)	40.6	2.6	40.7	2.4	40.9	2.4	0.068	35.6	2.6	35.8	2.4	35.8	2.6	<0.001

BMI, body mass index; KRW, Korean won; MET, metabolic equivalent; SMI, skeletal muscle index. Data are presented as means ± standard deviations (SD) or *n* (%). ^a^ Protein intake from milk, yogurt, and cheese. ^b^ *p* for trend was calculated from a linear regression analysis for continuous variables and Mantel-Haenszel x^2^ for categorical variables.

**Table 2:** Nutrient intakes of the study participants according to tertiles of dairy protein intake at baseline.

Variables	Men (*n* = 2096)							Women (*n* = 2316)						
	T1		T2		T3		*p* for Trend	T1		T2		T3		*p* for Trend
	Mean	SE	Mean	SE	Mean	SE		Mean	SE	Mean	SE	Mean	SE	
Energy (kcal/day)	1852.5	20.5	2008.3	20.8	2171.0	20.5	<0.001	1714.7	20.9	1861.2	21.3	2049.8	21.0	<0.001
Macronutrients (% of energy)														
Fat	13.9	0.2	16.1	0.2	17.3	0.2	<0.001	11.1	0.2	14.1	0.2	16.1	0.2	<0.001
Carbohydrates	71.6	0.2	68.9	0.2	67.5	0.2	<0.001	75.1	0.2	71.4	0.2	69.0	0.2	<0.001
Protein	13.1	0.1	13.9	0.1	14.3	0.1	<0.001	12.5	0.1	13.6	0.1	14.1	0.1	<0.001
Protein (g/day)	61.1	0.9	70.0	0.9	77.9	0.9	<0.001	53.7	0.8	63.3	0.8	72.6	0.8	<0.001
Dairy protein ^a^ (g/day)	0.1	0.1	2.0	0.1	8.0	0.1	<0.001	0.2	0.1	2.8	0.1	9.0	0.1	<0.001
Dairy protein (% of protein)	0.2	0.1	3.1	0.1	10.7	0.1	<0.001	0.4	0.2	4.9	0.2	13.2	0.2	<0.001
Dairy products (g/day)														
Milk	1.9	3.3	35.4	3.3	161.1	3.3	<0.001	2.7	2.4	43.2	2.4	194.2	2.4	<0.001
Yogurt	1.2	1.6	18.2	1.4	63.7	1.6	<0.001	2.5	1.7	30.8	1.8	62.8	1.8	<0.001
Cheese	0.0	0.1	0.5	0.1	1.0	0.1	<0.001	0.1	0.1	0.4	0.1	1.3	0.1	<0.001
Vitamins														
Vitamin A (ug RE/day)	449.4	13.1	566.8	13.3	653.4	13.1	<0.001	400.3	13.2	499.2	13.4	616.7	13.3	<0.001
Vitamin C (mg/day)	105.6	3.0	122.5	3.0	132.5	3.0	<0.001	115.0	3.5	137.0	3.5	144.0	3.5	<0.001
Vitamin E (mg/day)	8.4	0.2	9.8	0.2	10.7	0.2	<0.001	7.6	0.2	9.4	0.2	10.3	0.2	<0.001

RI, Recommended intake; RE, Retinal equivalent. All values were presented as adjusted means ± standard errors (SE) after adjusting for age and total energy intake (except energy intake) using a generalized linear model and were significantly different between the lowest and highest tertiles. ^a^ Protein intakes from milk, yogurt, and cheese.

**Table 3:** Hazard ratios (HRs) and 95% confidence intervals (95% CIs) for the risk of developing a low SMI according to tertiles of dairy protein intake at baseline.

Variables	Men (*n* = 2096)					Women (*n* = 2316)				
	T1	T2	T3	*p* for Trend	*p* for Interaction ^d^	T1	T2	T3	*p* for Trend	*p* for Interaction
Dairy protein intake ^a^ (g/day)										
Cases (n)/person-months	72/98,000	58/95,286	47/98,270			94/108,607	57/105,387	67/108,196		
HR (95% CI) ^b^	Reference	0.79 (0.54,1.15)	0.63 (0.42,0.94)	0.029	0.004	Reference	0.78 (0.54,1.12)	0.89 (0.63,1.28)	0.667	0.402
Higher protein intake ^c^										
Cases (n)/person-months	17/29,342	22/43,739	31/61,273			19/35,229	23/53,131	37/76,721		
HR (95% CI)	Reference	0.67 (0.34,1.35)	0.62 (0.32,1.19)	0.943		Reference	0.84 (0.47,1.51)	0.96 (0.57,1.64)	0.636	
Lower protein intake										
Cases (n)/person-months	55/68,658	36/51,547	16/36,997			75/73,378	34/52,256	30/31,475		
HR (95% CI)	Reference	0.84 (0.53, 1.31)	0.59 (0.35,0.99)	0.036		Reference	0.72 (0.45,1.15)	0.83 (0.50,1.36)	0.743	
Dairy protein intake (% of protein)										
Cases (n)/person-months	75/96,885	57/97,370	45/97,301			91/106,787	59/107,590	68/107,813		
HR (95% CI)	Reference	0.72 (0.49,1.04)	0.60 (0.41,0.89)	0.017	0.007	Reference	0.80 (0.55,1.15)	0.89 (0.63,1.26)	0.640	0.439
Higher protein intake										
Cases (n)/person-months	23/34,264	19/43,987	19/48,016			31/49,253	30/63,252	34/65,277		
HR (95% CI)	Reference	0.62 (0.32,1.19)	0.58 (0.30,1.12)	0.920		Reference	0.97 (0.56,1.69)	1.05 (0.61,1.81)	0.704	
Lower protein intake										
Cases (n)/person-months	52/62,621	38/53,383	26/49,285			60/57,534	29/44,338	34/42,536		
HR (95% CI)	Reference	0.78 (0.50,1.24)	0.56 (0.34,0.92)	0.010		Reference	0.69 (0.42,1.14)	0.77 (0.49,1.23)	0.458	
Milk protein intake (g/day)										
Cases (n)/person-months	81/107,429	55/94,575	41/89,552			98/114,331	46/83,691	74/124,168		
HR (95% CI)	Reference	0.86 (0.60,1.24)	0.66 (0.45,0.99)	0.048		Reference	0.78 (0.54,1.14)	0.88 (0.63,1.13)	0.641	
Yogurt protein intake (g/day)										
Cases (n)/person-months	80/123,787	42/54,157	55/113,612			55/113,612	86/120,518	46/66,918		
HR (95% CI)	Reference	1.28 (0.86,1.91)	0.74 (0.51,1.07)	0.037		Reference	0.90 (0.62,1.32)	0.96 (0.69,1.33)	0.930	

^a^ Protein intakes from milk, yogurt, and cheese. ^b^ Adjusted for baseline age (years), skeletal muscle mass at baseline, energy-adjusted protein intake (g/day), vitamins intake (sum of vitamin score: the first quartile received a score of 1; the second, third, and fourth quartiles received scores of 0), marital status (married/others), education level (≥college/others), income (≥3,000,000 KRW per month/other), smoking status (yes/no), alcohol consumption (yes/no), regular physical activity (METs-hours/day), self-perceived dental health status (poor/others), chronic diseases (yes/no), and residential area (urban/rural). ^c^ Higher protein intake: a total protein intake of ≥1.0 g/kg body weight/day; lower protein intake: a total protein intake of <1.0 g/kg body weight/day. ^d^ Test for interaction between protein intake and dairy protein intake.

**Table 4:** Hazard ratios (HRs) and 95% confidence intervals (95% CIs) for the risk of developing a low SMI according to milk and yogurt consumption frequencies at baseline.

Variables	Dairy Product Consumption (Servings) ^b^			
	<1/week	1–6/week	≥1/day	*p* for Trend
Milk Consumption frequency				
Men (*n* = 2096)				
Cases (n)/person-months	113/152,767	40/86,179	24/52,610	0.023
HR (95% CI) ^a^	Reference	0.68(0.47,0.99)	0.62 (0.39,0.98)	
Women (*n* = 2316)				
Cases (n)/person-months	125/151,642	37/89,864	56/80,684	0.657
HR (95% CI)	Reference	0.65 (0.44,0.96)	1.05 (0.75,1.48)	
Yogurt Consumption frequency				
Men (*n* = 2096)				
Cases (n)/person-months	121/177,375	40/83,213	16/30,968	
HR (95% CI)	Reference	0.70 (0.48,1.02)	0.70 (0.41,1.23)	0.161
Women (*n* = 2316)				
Cases (n)/person-months	131/186,333	58/95,677	29/40,180	
HR (95% CI)	Reference	0.96 (0.68,1.35)	1.11 (0.73,1.70)	0.996

^a^ Adjusted for baseline age (years), skeletal muscle mass at baseline, energy-adjusted protein intake (g/day), vitamins intake (sum of vitamin score: the first quartile received a score of 1; the second, third, and fourth quartiles received scores of 0), marital status (married/others), education level (≥college/others), income (≥3,000,000 KRW per month/other), smoking status (yes/no), alcohol consumption (yes/no), regular physical activity (METs-hours/day), self-perceived dental health status (poor/others), chronic diseases (yes/no), and residential area (urban/rural). ^b^ One serving was equal to 200 mL of milk, 130 mL of yogurt, and 20 g of cheese.

## References

[1] Cruz-Jentoft A.J., Bahat G., Bauer J., Boirie Y., Bruyère O., Cederholm T., Cooper C., Landi F., Rolland Y., Sayer A.A. (2019). Sarcopenia: Revised European consensus on definition and diagnosis. Age Ageing.

[2] Rom O., Kaisari S., Aizenbud D., Reznick A.Z. (2012). Lifestyle and sarcopenia-etiology, prevention, and treatment. Rambam Maimonides Med. J..

[3] Beard J., Officer A., Cassels A. (2016). World Report on Ageing and Health.

[4] Keller K., Engelhardt M. (2013). Strength and muscle mass loss with aging process. Age and strength loss. Muscles Ligaments Tendons J..

[5] Burton L.A., Sumukadas D. (2010). Optimal management of sarcopenia. Clin. Interv. Aging.

[6] Beasley J.M., Shikany J.M., Thomson C.A. (2013). The role of dietary protein intake in the prevention of sarcopenia of aging. Nutr. Clin. Pract..

[7] Hulmi J.J., Lockwood C.M., Stout J.R. (2010). Effect of protein/essential amino acids and resistance training on skeletal muscle hypertrophy: A case for whey protein. Nutr. Metab..

[8] Dong J.Y., Zhang L., He K., Qin L.Q. (2011). Dairy consumption and risk of breast cancer: A meta-analysis of prospective cohort studies. Breast Cancer Res. Treat..

[9] Larsson S.C., Andersson S.-O., Johansson J.-E., Wolk A. (2008). Cultured milk, yogurt, and dairy intake in relation to bladder cancer risk in a prospective study of Swedish women and men. Am. J. Clin. Nutr..

[10] Dehghan M., Mente A., Rangarajan S., Sheridan P., Mohan V., Iqbal R., Gupta R., Lear S., Wentzel-Viljoen E., Avezum A. (2018). Association of dairy intake with cardiovascular disease and mortality in 21 countries from five continents (PURE): A prospective cohort study. Lancet.

[11] Shin S., Lee H.W., Kim C.E., Lim J., Lee J.K., Kang D. (2017). Association between milk consumption and Metabolic Syndrome among Korean adults: Results from the health examinees study. Nutrients.

[12] Kim D., Kim J. (2017). Dairy consumption is associated with a lower incidence of the metabolic syndrome in middle-aged and older Korean adults: The Korean Genome and Epidemiology Study (KoGES). Br. J. Nutr..

[13] Kesse-Guyot E., Assmann K., Andreeva V., Ferry M., Hercberg S., Galan P. (2016). Consumption of dairy products and cognitive functioning: Findings from the SU. VI. MAX 2 study. J. Nutr. Health Aging.

[14] Cuesta-Triana F., Verdejo-Bravo C., Fernandez-Perez C., Martin-Sanchez F.J. (2019). Effect of milk and other dairy products on the risk of frailty, sarcopenia, and cognitive performance decline in the elderly: A systematic review. Adv. Nutr..

[15] Hess J.M., Jonnalagadda S.S., Slavin J.L. (2016). Dairy Foods: Current evidence of their effects on bone, cardiometabolic, cognitive, and digestive health. Compr. Rev. Food Sci. Food Saf..

[16] Sato Y., Iki M., Fujita Y., Tamaki J., Kouda K., Yura A., Moon J.S., Winzenrieth R., Iwaki H., Ishizuka R. (2015). Greater milk intake is associated with lower bone turnover, higher bone density, and higher bone microarchitecture index in a population of elderly Japanese men with relatively low dietary calcium intake: Fujiwara-kyo osteoporosis risk in men (FORMEN) study. Osteoporos. Int..

[17] Sahni S., Tucker K.L., Kiel D.P., Quach L., Casey V.A., Hannan M.T. (2013). Milk and yogurt consumption are linked with higher bone mineral density but not with hip fracture: The Framingham Offspring Study. Arch. Osteoporos..

[18] Bonjour J.-P., Kraenzlin M., Levasseur R., Warren M., Whiting S. (2013). Dairy in adulthood: From foods to nutrient interactions on bone and skeletal muscle health. J. Am. Coll. Nutr..

[19] Shin S., Hong K., Kang S.W., Joung H. (2013). A milk and cereal dietary pattern is associated with a reduced likelihood of having a low bone mineral density of the lumbar spine in Korean adolescents. Nutr. Res..

[20] Houston D.K., Stevens J., Cai J., Haines P.S. (2005). Dairy, fruit, and vegetable intakes and functional limitations and disability in a biracial cohort: The Atherosclerosis Risk in Communities Study. Am. J. Clin. Nutr..

[21] Machado-Fragua M.D., Struijk E.A., Caballero F.F., Ortola R., Lana A., Banegas J.R., Rodriguez-Artalejo F., Lopez-Garcia E. (2020). Dairy consumption and risk of falls in 2 European cohorts of older adults. Clin. Nutr..

[22] Hanach N.I., McCullough F., Avery A. (2019). The Impact of Dairy Protein Intake on Muscle Mass, Muscle Strength, and Physical Performance in Middle-Aged to Older Adults with or without Existing Sarcopenia: A Systematic Review and Meta-Analysis. Adv. Nutr..

[23] Radavelli-Bagatini S., Zhu K., Lewis J.R., Dhaliwal S.S., Prince R.L. (2013). Association of dairy intake with body composition and physical function in older community-dwelling women. J. Acad. Nutr. Diet..

[24] Alexandrov N.V., Eelderink C., Singh-Povel C.M., Navis G.J., Bakker S.J.L., Corpeleijn E. (2018). Dietary protein sources and muscle mass over the life course: The lifelines cohort study. Nutrients.

[25] Alemán-Mateo H., Macías L., Esparza-Romero J., Astiazaran-García H., Blancas A.L. (2012). Physiological effects beyond the significant gain in muscle mass in sarcopenic elderly men: Evidence from a randomized clinical trial using a protein-rich food. Clin. Interv. Aging.

[26] Björkman M.P., Pilvi T., Kekkonen R., Korpela R., Tilvis R. (2011). Similar effects of leucine rich and regular dairy products on muscle mass and functions of older polymyalgia rheumatica patients: A randomized crossover trial. J. Nutr. Health Aging.

[27] Lee J.H., Lee H.S., Kim H., Kwon Y.J., Lee J.W. (2019). Association of milk consumption frequency on muscle mass and strength: An analysis of three representative Korean population studies. Eur. J. Nutr..

[28] DeSalvo K.B., Olson R., Casavale K.O. (2016). Dietary guidelines for Americans. JAMA.

[29] Drummond M.J., Rasmussen B.B. (2008). Leucine-enriched nutrients and the regulation of mTOR signalling and human skeletal muscle protein synthesis. Curr. Opin. Clin. Nutr. Metab. Care.

[30] Phillips S.M., Tang J.E., Moore D.R. (2009). The role of milk-and soy-based protein in support of muscle protein synthesis and muscle protein accretion in young and elderly persons. J. Am. Coll. Nutr..

[31] Montagnese C., Santarpia L., Buonifacio M., Nardelli A., Caldara A.R., Silvestri E., Contaldo F., Pasanisi F. (2015). European food-based dietary guidelines: A comparison and update. Nutrition.

[32] Chaltiel D., Adjibade M., Deschamps V., Touvier M., Hercberg S., Julia C., Kesse-Guyot E. (2019). Programme national nutrition sante-guidelines score 2 (PNNS-GS2): Development and validation of a diet quality score reflecting the 2017 French dietary guidelines. Br. J. Nutr..

[33] Yoshiike N., Hayashi F., Takemi Y., Mizoguchi K., Seino F. (2007). A new food guide in Japan: The Japanese food guide spinning top. Nutr. Rev..

[34] Ministry of Health and Welfare, The Korean Nutrition Society (2015). Dietary Reference Intakes for Koreans 2015.

[35] Jun S., Ha K., Chung S., Joung H. (2016). Meat and milk intake in the rice-based Korean diet: Impact on cancer and metabolic syndrome. Proc. Nutr. Soc..

[36] Korea Centers for Disease Control and Prevention (2016). Korea Health Statistics 2015.

[37] Michaelsson K., Wolk A., Langenskiold S., Basu S., Warensjo Lemming E., Melhus H., Byberg L. (2014). Milk intake and risk of mortality and fractures in women and men: Cohort studies. BMJ.

[38] So E., Choi S.K., Joung H. (2019). Impact of dietary protein intake and obesity on lean mass in middle-aged individuals after a 12-year follow-up: The Korean Genome and Epidemiology Study (KoGES). Br. J. Nutr..

[39] Ahn Y., Kwon E., Shim J.E., Park M.K., Joo Y., Kimm K., Park C., Kim D.H. (2007). Validation and reproducibility of food frequency questionnaire for Korean genome epidemiologic study. Eur. J. Clin. Nutr..

[40] The Korean Nutrition Society (2000). Food composition table. Recommended Dietary Allowances for Koreans.

[41] Bauer J., Biolo G., Cederholm T., Cesari M., Cruz-Jentoft A.J., Morley J.E., Phillips S., Sieber C., Stehle P., Teta D. (2013). Evidence-based recommendations for optimal dietary protein intake in older people: A position paper from the PROT-AGE Study Group. J. Am. Med. Dir. Assoc..

[42] World Health Organization (2010). Global Recommendations on Physical Activity for Health.

[43] Nelson M.E., Rejeski W.J., Blair S.N., Duncan P.W., Judge J.O., King A.C., Macera C.A., Castaneda-Sceppa C. (2007). Physical activity and public health in older adults: Recommendation from the American College of Sports Medicine and the American Heart Association. Med. Sci. Sports Exerc..

[44] Ainsworth B.E., Haskell W.L., Whitt M.C., Irwin M.L., Swartz A.M., Strath S.J., O’brien W.L., Bassett D.R., Schmitz K.H., Emplaincourt P.O. (2000). Compendium of physical activities: An update of activity codes and MET intensities. Med. Sci. Sports Exerc..

[45] Clarys J., Martin A., Drinkwater D. (1984). Gross tissue weights in the human body by cadaver dissection. Hum. Biol..

[46] Janssen I., Heymsfield S.B., Ross R. (2002). Low relative skeletal muscle mass (sarcopenia) in older persons is associated with functional impairment and physical disability. J. Am. Geriatr. Soc..

[47] Kim Y.S., Lee Y., Chung Y.S., Lee D.J., Joo N.S., Hong D., Song G., Kim H.J., Choi Y.J., Kim K.M. (2012). Prevalence of sarcopenia and sarcopenic obesity in the Korean population based on the Fourth Korean national health and nutritional examination surveys. J. Gerontol. A Biol. Sci. Med. Sci..

[48] Willett W.C., Howe G.R., Kushi L.H. (1997). Adjustment for total energy intake in epidemiologic studies. Am. J. Clin. Nutr..

[49] Bartali B., Curto T., Maserejian N., Araujo A. (2015). Intake of antioxidants and subsequent decline in physical function in a racially/ethnically diverse population. J. Nutr. Health Aging.

[50] Aleman-Mateo H., Carreon V.R., Macias L., Astiazaran-Garcia H., Gallegos-Aguilar A.C., Enriquez J.R. (2014). Nutrient-rich dairy proteins improve appendicular skeletal muscle mass and physical performance, and attenuate the loss of muscle strength in older men and women subjects: A single-blind randomized clinical trial. Clin. Interv. Aging.

[51] Abargouei A.S., Janghorbani M., Salehi-Marzijarani M., Esmaillzadeh A. (2012). Effect of dairy consumption on weight and body composition in adults: A systematic review and meta-analysis of randomized controlled clinical trials. Int. J. Obes..

[52] Verdijk L.B., Jonkers R.A., Gleeson B.G., Beelen M., Meijer K., Savelberg H.H., Wodzig W.K., Dendale P., van Loon L.J. (2009). Protein supplementation before and after exercise does not further augment skeletal muscle hypertrophy after resistance training in elderly men. Am. J. Clin. Nutr..

[53] Tieland M., van de Rest O., Dirks M.L., van der Zwaluw N., Mensink M., van Loon L.J., de Groot L.C. (2012). Protein supplementation improves physical performance in frail elderly people: A randomized, double-blind, placebo-controlled trial. J. Am. Med. Dir. Assoc..

[54] Campbell W.W., Leidy H.J. (2007). Dietary protein and resistance training effects on muscle and body composition in older persons. J. Am. Coll. Nutr..

[55] Wolfe R.R. (2015). Update on protein intake: Importance of milk proteins for health status of the elderly. Nutr. Rev..

[56] Fulgoni V.L., Keast D.R., Auestad N., Quann E.E. (2011). Nutrients from dairy foods are difficult to replace in diets of Americans: Food pattern modeling and an analyses of the National health and nutrition examination survey 2003–2006. Nutr. Res..

[57] Lim H., Kim S.Y., Wang Y., Lee S.J., Oh K., Sohn C.Y., Moon Y.M., Jee S.H. (2014). Preservation of a traditional Korean dietary pattern and emergence of a fruit and dairy dietary pattern among adults in South Korea: Secular transitions in dietary patterns of a prospective study from 1998 to 2010. Nutr. Res..

[58] Park H.A. (2018). Adequacy of Protein Intake among Korean Elderly: An Analysis of the 2013–2014 Korea National Health and Nutrition Examination Survey Data. Korean J. Family Med..

[59] Du Y., Oh C., No J. (2019). Advantage of dairy for improving aging muscle. J. Obes. Metab. Syndr..

[60] Lana A., Rodriguez-Artalejo F., Lopez-Garcia E. (2015). Dairy consumption and risk of frailty in older adults: A prospective cohort study. J. Am. Geriatr. Soc..

[61] Zemel M., Richards J., Mathis S., Milstead A., Gebhardt L., Silver E. (2005). Dairy augmentation of total and central fat loss in obese subjects. Int. J. Obes..

[62] Yoo J.-I., Ha Y.-C., Lee Y.-K., Yoo M.-J., Koo K.-H. (2017). High prevalence of sarcopenia among binge drinking elderly women: A nationwide population-based study. BMC Geriatr..

[63] Kim K.M., Jang H.C., Lim S. (2016). Differences among skeletal muscle mass indices derived from height-,weight-, and body mass index-adjusted models in assessing sarcopenia. Korean J. Intern. Med..

[64] Liochev S.I. (2013). Reactive oxygen species and the free radical theory of aging. Free Radic. Biol. Med..

[65] Kim J.-S., Wilson J.M., Lee S.-R. (2010). Dietary implications on mechanisms of sarcopenia: Roles of protein, amino acids and antioxidants. J. Nutr. Biochem..

[66] Chaput J., Lord C., Cloutier M., Aubertin-Leheudre M. (2007). Relationship between antioxidant intakes and class I sarcopenia in elderly men and women. J. Nutr. Health Aging.

